# Oxidized LDL Is Strictly Limited to Hyperthyroidism Irrespective of Fat Feeding in Female Sprague Dawley Rats

**DOI:** 10.3390/ijms160511689

**Published:** 2015-05-21

**Authors:** Sieglinde Zelzer, Harald Mangge, Sabine Pailer, Herwig Ainoedhofer, Petra Kieslinger, Tatjana Stojakovic, Hubert Scharnagl, Florian Prüller, Daniel Weghuber, Christian Datz, Johannes Haybaeck, Barbara Obermayer-Pietsch, Christian Trummer, Johanna Gostner, Hans-Jürgen Gruber

**Affiliations:** 1 Clinical Institute of Medical and Chemical Laboratory Diagnostics, Medical University Graz, 8036 Graz, Austria; E-Mails: sieglinde.zelzer@klinikum-graz.at (S.Z.); sabine.pailer@klinikum-graz.at (S.P.); herwig.ainoedhofer@klinikum-graz.at (H.A.); petra.kieslinger@klinikum-graz.at (P.K.); tatjana.stojakovic@medunigraz.at (T.S.); hubert.scharnagl@medunigraz.at (H.S.); florian.prueller@klinikum-graz.at (F.P.); hans.gruber@medunigraz.at (H.-J.G.); 2 Department of Pediatrics, Paracelsus Medical University Salzburg, 5020 Salzburg, Austria; E-Mail: d.weghuber@salk.at; 3 Obesity Research Unit, Paracelsus Medical University and Salzburger Landeskliniken, 5020 Salzburg, Austria; E-Mail: c.datz@kh-oberndorf.at; 4 Department of Internal Medicine, General Hospital Oberndorf, 5110 Oberndorf, Austria; 5 Department of Pathology, Medical University of Graz, 8036 Graz, Austria; E-Mail: johannes.haybaeck@medunigraz.at; 6 Division of Endocrinology and Metabolism, Medical University Graz, 8036 Graz, Austria; E-Mails: barbara.obermayer@medunigraz.at (B.O.-P.); christian.trummer@klinikum-graz.at (C.T.); 7 Medical Biochemistry, Center of Chemistry and Biomedicine, Medical University of Innsbruck, 6020 Innsbruck, Austria; E-Mail: johanna.gostner@i-med.ac.at

**Keywords:** hyperthyroidism, oxidized LDL, hypothyroidism, fat feeding, metabolic dysfunction, Sprague Dawley rats

## Abstract

Metabolic dysfunctions might play a crucial role in the pathophysiology of thyroid dysfunctions. This study aimed to investigate the impact of a controlled diet (normal *versus* high fat feeding) on hypothyroid and hyperthyroid Sprague Dawley rats. Female Sprague Dawley rats (*n* = 66) were grouped into normal diet (*n* = 30) and high-fat diet (*n* = 36) groups and subdivided into controls, hypothyroid and hyperthyroid groups, induced through propylthiouracil or triiodothyronine (T3) treatment, respectively. After 12 weeks of treatment metabolic parameters, such as oxidized LDL (oxLDL), malondialdehyde (MDA), 4-hydroxynonenal (HNE), the lipid profile, body weight and food intake parameters were analyzed. Successfully induced thyroid dysfunctions were shown by T3 levels, both under normal and high fat diet. Thyroid dysfunctions were accompanied by changes in calorie intake and body weight as well as in the lipid profile. In detail, hypothyroid rats showed significantly decreased oxLDL levels, whereas hyperthyroid rats showed significantly increased oxLDL levels. These effects were seen under high fat diet and were less pronounced with normal feeding. Taken together, we showed for the first time in female SD rats that only hyper-, but not hypothyroidism, is associated with high atherogenic oxidized LDL irrespective of normal or high-fat diet in Sprague Dawley rats.

## 1. Introduction

Thyroid dysfunctions comprise a complex network of diseases including malignant, inflammatory and autoimmune diseases of the thyroid gland [1,2,3]. Functionally, thyroid diseases can by divided into hypo- and hyperthyroidism, according to thyroid hormone production, e.g., triiodothyronine (T3) [4]. There is established evidence that thyroid dysfunctions such as hypo- and hyperthyroidism as well as subclinical hypothyroidism are associated with metabolic dysfunctions, especially regarding dyslipidemia [5,6]. Hence, hypothyroidism is associated with high levels of total and LDL-cholesterol, and hyperthyroidism with low levels of both. Associations with other lipid parameters, such as triglycerides, HDL cholesterol, and various lipoproteins with thyroid dysfunctions, are a matter of debate. Higher rates in both hypo- and hyperthyroidism have been reported [7]. Beyond dyslipidemia, thyroid hormones are associated with radical oxygen species (ROS) production due to metabolic rate effects. ROS modify endothelial functions by a variety of mechanisms, such as peroxidation of membrane lipids, which contributes to metabolic and cardiovascular risk [7,8,9]. The production of ROS contributes to atherogenic oxidized LDL, which further advances metabolic and cardiovascular risk. Nevertheless, findings especially regarding hypothyroidism and ROS are controversial to date. Reduced [7], increased [10] as well as unchanged [11] ROS levels are described. Otherwise, hyperthyroidism implies an increase of ROS, which seems to be correlated with thyroid hormone overproduction.

We hypothesized that thyroid dysfunctions, both hypo- and hyperthyroidisms, may be associated with altered oxidative stress which contributes to lipid peroxidation and an atherogenic lipid profile. Moreover, effects of a high-fat diet on these mechanisms may play a role. Thus, we aimed to investigate the effects of thyroid dysfunctions on ROS production and the lipid profile under normal and a high-fat diet in Sprague Dawley rats.

## 2. Results

Successful induction of thyroid dysfunction was determined via T3 showing highly significant increased T3 levels up to two to three-fold in hyperthyroid groups and markedly decreased levels in hypothyroid groups, both under normal and high-fat diet (HFD), Table 1. Analyses of body weight changes and food consumption revealed that hyperthyroid rats compared to appropriate controls showed body weight changes between 12% and 19% (Supplementary Figure S1), and increased food consumption both regarding food intake and calorie intake as shown in Table 1. These effects are seen under normal as well as under HFD. In contrast, induction of hypothyroidism reduced body weights and food consumption compared to appropriate controls. In detail, with the normal diet up to −13% reduction of body weight, and about −50% of food intake were seen, which resulted in a decreased calorie uptake of −47%. These effects are also seen under HFD. To analyze the effects of high-fat diet and thyroid dysfunctions lipid profiles were determined.

### 2.1. Triglycerides, Free Fatty Acids, Phospholipids, HDL, Cholesterol

As shown in Table 1, triglycerides and free fatty acids were significantly decreased in the hypothyroid rats compared to controls in both diet groups. Interestingly, HDL levels were significantly increased in the HFD hypothyroid group (Table 1). No statistically significant differences were seen in the hyperthyroid groups concerning triglycerides, free fatty acids and HDL. Analyses of cholesterol and free cholesterol showed significantly decreased levels between hyperthyroid rats and controls under normal diet. Hypothyroid rats under HFD had significantly increased levels (Table 1).

### 2.2. Malondialdehyde (MDA), 4-Hydroxynonenal (HNE)

Analyses of MDA revealed significant differences regarding thyroid dysfunctions and diet (Table 1). Firstly, we found significantly reduced MDA levels in HFD controls compared to normal diet controls. Hypothyroid rats under normal diet showed no differences compared to appropriate controls whereas hyperthyroid rats under normal diet showed highly significant reduced MDA levels. In contrast, HFD hypothyroid rats showed highly significant reduced MDA levels whereas hyperthyroid rats showed no differences compared to HFD controls, as shown in detail in Table 1. Analyses of HNE showed a not statistical significant increase of HNE in hyperthyroid rats in both diet groups of about 35%, whereas no differences were seen in hypothyroid rats, again in both groups.

### 2.3. Oxidized Low Dense Lipoprotein (oxLDL)

**Figure 1:**
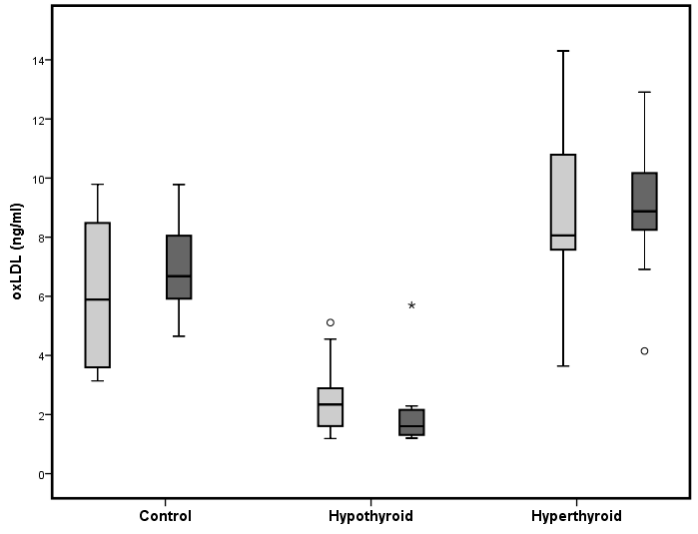
Box and Whisker Blot of oxidized LDL. Grey bars indicate normal diet and black bars indicate high fat diet. * *p* < 0.05 compared to appropriate diet control group; ^º^ *p* < 0.05 compared to normal diet control group.

Atherogenic oxLDL was significantly increased in hyperthyroid rats, both under normal and HFD, compared to appropriate controls (Table 1). Hypothyroid rats showed a highly significant reduction of oxLDL compared to appropriate controls. These effects are more pronounced under HFD. Furthermore, comparing untreated controls under normal diet and high-fat diet to determine effects of HFD on oxLDL revealed no statistically significant differences (Figure 1).

**Table 1:** Characteristics of female SD rats after a 12-week treatment.

	Normal Diet Group (*n* = 30)			High Fat Diet Group (*n* = 36)		
	Control (*n* = 10)	Hypothyroid (*n* = 10)	Hyperthyroid (*n* = 10)	Control (*n* = 10)	Hypothyroid (*n* = 10)	Hyperthyroid (*n* = 16)
Body weight change (g)	36 ± 24	−35 ± 8 ***	31 ± 7	56 ± 24	−42 ± 16 ***	49 ± 17 ^††^
Body weight change (%)	14 ± 10	−13 ± 2 ***	12 ± 3	21 ± 9	−15 ± 5 ***	19 ± 7
Food intake/rate/day (g)	19	10	30	13	8	18
Calorie intake/rat/day (kcal)	49	26	80	61	38	87
T3 (pg/mL)	417 ± 85	269 ± 74 **	951 ± 454 **	450 ± 58	446 ± 176 ^††^	1708 ± 1447
Oxidized LDL (ng/mL)	6.1 ± 2.5	2.6 ± 1.3 **	8.8 ± 3.0 *	6.9 ± 1.6	2.1 ± 1.3 ***	9.5 ± 3.0 *
MDA (nmol/mL)	1.4 ± 0.2	1.4 ± 0.3	1.0 ± 0.2 ***	1.0 ± 0.1 ^ººº^	0.7 ± 0.1 ^†††^	1.0 ± 0.3
HNE (nmol/mL)	0.09 ± 0.02	0.09 ± 0.03	0.12 ± 0.05	0.08 ± 0.02	0.07 ± 0.02	0.11 ± 0.04
Triglyceride (mg/dL)	62 ± 8	46 ± 4 ***	61 ± 36	50 ± 12 ^º^	32 ± 6 ^†††^	88 ± 14
Cholesterol (mg/dL)	86 ± 13	87 ± 14	69 ± 17 *	81 ± 14	185 ± 23 ^†††^	64 ± 13
Free cholesterol (mg/dL)	24 ± 4	26 ± 5	19 ± 6 *	20 ± 5	59 ± 8 ^†††^	18 ± 7
Phospholipids (mg/dL)	156 ± 15	120 ± 19 **	141 ± 21	139 ± 22	209 ± 21 ^†††^	129 ± 26
Free fatty acids (mmol/L)	1.1 ± 0.2	0.7 ± 0.2 **	1.0 ± 0.3	0.8 ± 0.1 ^ºº^	0.5 ± 0.1	1.2 ± 0.5
HDL cholesterol (mg/dL)	44 ± 5	39 ± 6	41 ± 7	44 ± 5	66 ± 5 ^†††^	40 ± 9

MDA: malondialdehyde; HNE: 4-hydroxynonenal; Data are presented as means ± standard deviations * *p* < 0.05, ** *p* < 0.01, *** *p* < 0.001 compared to appropriate control; ^º^ *p* < 0.05, ^ºº^ *p* < 0.01, ^ººº^ *p* < 0.001 compared to normal diet control group; ^††^ *p* < 0.01, ^†††^ *p* < 0.001 compared to appropriate normal diet group.

We subsequently investigated the association of thyroid dysfunctions with lipid profile changes by linear regression analyses. We found an association of T3 with oxLDL in both untreated control groups, with *r* = 0.702 and *p* = 0.024 under HFD and a not statistically significant association in the normal diet group with *r* = 0.630 and *p* = 0.051 (not shown). Other lipid parameters, including the oxidative stress parameters such as MDA and HNE, showed no significant correlations with thyroid dysfunctions.

## 3. Discussion

We thus show that both hyper- and hypothyroidism is associated with significantly altered oxLDL levels in female Sprague Dawley rats. Hence, oxLDL was significantly increased in hyper-, and significantly decreased in hypothyroid rats. Interestingly, we found no significant additive effects of a high-fat diet on oxLDL levels. To the best of our knowledge, we are the first to show this, and to investigate lipid profiles together with a broad spectrum of oxidative “stress” markers in rats with thyroid dysfunctions subjected to various fat-feeding conditions.

Even though, some clinical studies have suggested a stronger LDL oxidation in hyper- as compared to hypo-, or normothyroid controls, the findings remain controversial [12,13]. Hence, the debate goes on, and increased, decreased, or even unaltered oxidative stress markers including MDA and HNE have been described in context with imbalanced thyroid function [7,8,11,14,15,16]. In normothyroid control rats that had a high-fat diet, we found significantly increased MDA levels compared to those with the normal diet. This indicates metabolic stress due to high fat and high caloric intake. Hypothyroidism reduced MDA levels in the high-fat diet group whereas no differences were seen under the normal diet. Hyperthyroid rats showed reduced MDA levels in the normal diet group, and no differences were seen under the high-fat diet. Interestingly HNE, as a marker of cell membrane lipid oxidation, showed no significant differences in any group.

Undoubtedly, as reviewed in detail by Villanueva *et al.* [10] substantial evidence exists that metabolic effects of thyroid hormones are directly linked to ROS production and oxidative stress in various ways, depending on the underlying pathophysiological mechanisms of thyroid disease, e.g., autoimmune disease, goiter, nodules or malign diseases. Regarding the functional status and thyroid hormone production, it is hypothesized that hyperthyroidism implies an increase in oxidative stress which is driven by the thyroid hormone overproduction rate, whereas hypothyroidism implies a reduction in the antioxidant activity and ROS production, which mainly results in reduced or unchanged oxidative stress. These effects include, among others, stimulation of substrate oxidation and mitochondrial oxygen consumption. Interestingly, this is in line with our findings regarding oxidized LDL and thyroid dysfunctions only.

Next, we investigated the influence of thyroid status on lipid profiles, especially in regard to a high-fat diet. Firstly, thyroid dysfunctions, both hypo- and hyperthyroidism, impacted the lipid profile significantly compared to untreated rats. In particular, we found reduced levels of triglycerides, phospholipids, and free fatty acids in hypothyroid rats, and reduced levels of cholesterol and free cholesterol in hyperthyroid rats. Under a high-fat diet containing 58% fat, the levels of cholesterol and free cholesterol increased highly significantly in hypothyroid rats. This was accompanied by a decrease of triglycerides. Interestingly, hyperthyroid rats with a high-fat diet did not show any significant differences in the lipid profile compared to appropriate high-fat controls. One may speculate that a higher metabolic rate in hyperthyroid rats drives these effects. Hence, for the first time in rats, we show robust significant differences in the lipid profile regarding thyroid status, which persist under normal and high fat diet.

Human studies found increased levels of cholesterol and triglycerides in hypothyroidism and reduced levels in hyperthyroidism [5,7,17,18] which partly contradicts our findings in rats. This may be explained by the fact that in rats experimental hypothyroidism induces cretinism as described in detail elsewhere [19]. Cretinism causes growth retardation and a strongly reduced metabolic rate which may be responsible for the different findings.

Thyroid hormone suppression in rats as a consequence of propylthiouracil treatment may induce side effects, e.g., hepatotoxicity. To avoid such side effects in this study, dose and duration of treatment were based on previous studies where the treatment was well tolerated [19,20,21,22].

Taken together, hypothyroidism in female Sprague Dawley rats is associated with low, and hyperthyroidism with strongly increased, oxLDL levels. This effect remains unchanged under both normal and high-fat diets. As increased levels of oxLDL contribute essentially to atherogenesis, our data underline a potentially strong impact of thyroid function on cardiovascular risk, at least in females.

## 4. Experimental Section

### 4.1. Animals

Female Sprague Dawley (SD) rats (Himberg, Austria), were housed (three per cage) and maintained on a 12 h light:12 h dark cycle in a temperature- (21 ± 2 °C) and humidity-controlled animal facility. The animals were acclimatized for one week under the same laboratory conditions of photoperiod, humidity and room-temperature. Rats initially weighing 251 ± 21 g were either assigned to normal rodent diet groups (Altromin, Germany) with 2.6 kcal/g and 11% fat or custom-designed high-fat diet (HFD) groups with 4.7 kcal/g and 56% fat (Harlan, Austria). The HFD composition was based on previous studies [23,24,25]. Food and tap water were provided *ad libitum*. Experimental setup for female SD rats was performed according to the guidelines of the Animal Care and Use Committee of the Ministry of Science and Research, Vienna, Austria and approved by the responsible national ethics committee.

### 4.2. Experimental Design and Treatment

The rats were randomly allocated into 6 groups as shown in Table 1. Groups 1–3 were fed a normal rodent diet, whereas groups 4–6 were fed with a HFD. Hypothyroidism was induced via administration of 6-propylthiouracil (0.04 g/100 mL) into the tap drinking water over the whole experimental period of 12 weeks as described [19,20,21]. Hyperthyroidism was induced with 3,3',5-triiodothyronine (T3) (300 μg/kg in 0.50 mM NaOH) via intraperitoneal (i.p.) injections every other day for 12 weeks [20].

### 4.3. Blood Collection

After 12 weeks, blood was obtained via heart puncture after an overnight fast. Rats were anesthetized with isolflurane (Forane, Abbott, Austria) prior to blood sampling. Blood was collected using S-Monovette^®^ Serum-Gel tubes (Sarstedt, Nürnbrecht, Germany) and centrifuged at ambient temperature. Samples were aliquoted and stored at −80 °C until analysis.

### 4.4. Laboratory Procedures

Total T3 and oxidized LDL levels were determined by commercial Rat ELISA (Uscn Life Science Inc., Wuhan, China). During the course of treatment, body weight measurements were performed at a 3-week interval and food consumption and calorie intake were recorded. The amount of ingested diet was calculated as the difference between the amount of food that was placed in the food bin and the weight of food that remained in the bin. Calorie intake per rat per day was calculated via food consumption per animal per day and the diets’ metabolizable energy values as kilocalories per gram (kcal/g). Determination of the oxidative stress markers malondialdehyde (MDA) and 4-hydroxynonenal (HNE) were based on GC-MS methods. MDA was measured using derivation with 2,4-dinitrophenylhydrazine. Ions were detected at *m*/*z* 204 for MDA and at *m*/*z* 206 for the deuterated analog (MDA-d_2_) as internal standard [26]. The HNE method is modified from Luo *et al.* [27], and based on derivatisation of HNE with pentafluorobenzylhydroxylamine-HCl followed by trimethylsilylation with *N*-methyl-*N*-(trimethylsilyl)-trifluoroacetamide. Representative ions in negative-ion chemical ionization mode were recorded at *m*/*z* 152 for HNE and at *m*/*z* 162 for the deuterated analogon (HNE-d11) as internal standard. Both markers were measured on a Thermo Trace GC Ultra coupled to a Thermo DSQ II mass spectrometer (Thermo Fisher Scientific, Waltham, MA, USA). Total cholesterol, free cholesterol, triacylglycerides, phospholipids, and HDL cholesterol (homogeneous assay) were measured using enzymatic reagents from Diasys (Holzheim, Germany) and were calibrated using secondary standards from Roche Diagnostics (Mannheim, Germany). Free fatty acids were measured using enzymatic reagents form Wako Chemicals (Neuss, Germany). All measurements were performed on an Olympus AU640 (Olympus, Hamburg, Germany) automatic analyzer.

### 4.5. Statistical Analysis

Data are presented as means ± standard deviations. Continuous variables were compared using Student’s *t*-test for independent samples or Mann-Whitney *U*-test depending on the distribution of data. Correlations between variables were determined by linear regression analysis according to Pearson (*r*, Pearson correlation coefficient; *p*, univariate ANOVA). *p*-values < 0.05 were considered statistically significant. Analyses were performed by explorative data analyses using SPSS for Windows (SPSS Inc., Chicago, IL, USA).

## Supplementary Materials

Supplementary materials can be found at http://www.mdpi.com/1422-0067/16/05/11689/s1.

## Highlights

To investigate lipid peroxidation a rat model was established. Hence, eu-, hypo- and hyperthyroid rats received normal and high-fat diets.

It is shown for the first time that:

(1) LDL oxidation is strictly driven by hyperthyroidism.
(2) The above effect persists independently of diet.
