# Supplementary material for: Oxidized LDL Is Strictly Limited to Hyperthyroidism Irrespective of Fat Feeding in Female Sprague Dawley Rats
Source: Int J Mol Sci. 2015 May 21;16(5):11689–98. doi: 10.3390/ijms160511689 (PMC4463724; doi:10.3390/ijms160511689)
Supplement: Supplementary file 1 [file ijms-16-11689-s001.pdf]

Supplementary Information

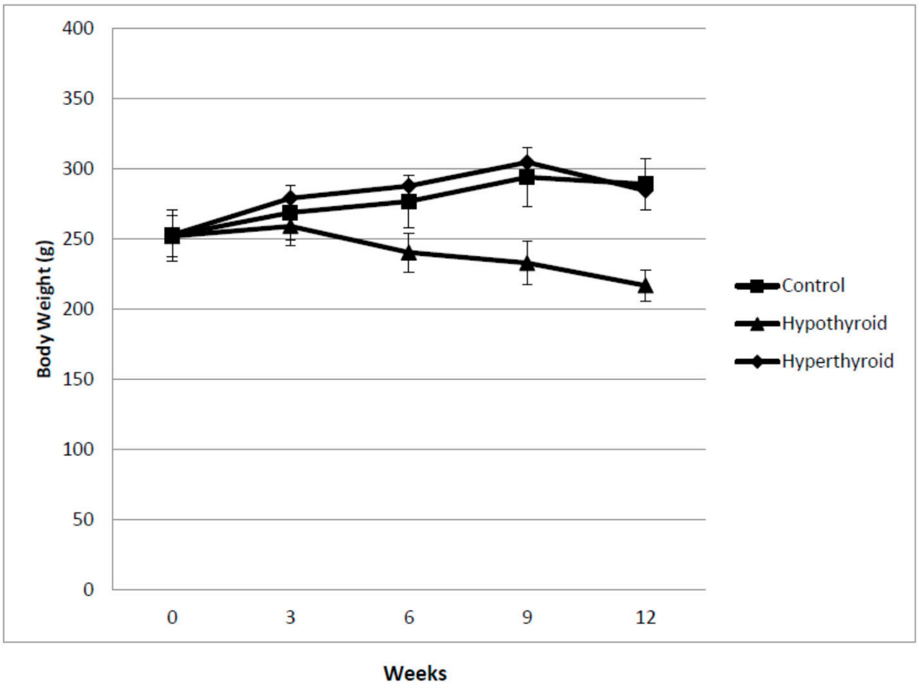

(**A**)

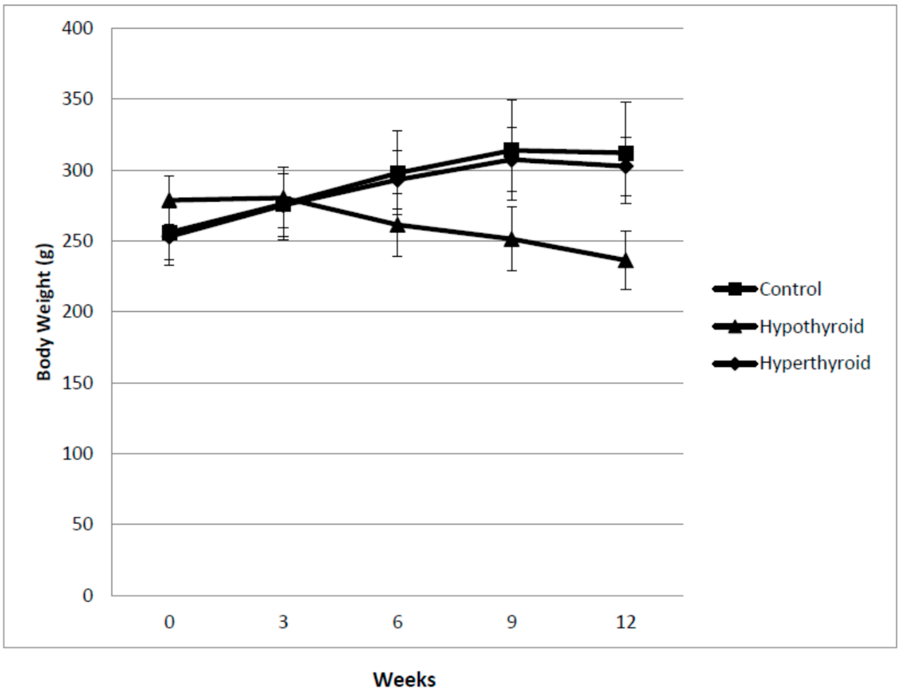

(**B**)

Figure S1. Body weight changes under (**A**) normal diet and (**B**) high fat diet.
